# Growth, Survival, and Intestinal Health Alterations in Mediterranean Yellowtail (*Seriola dumerili*) Due to Alternatives to Fishmeal and Fish Oil

**DOI:** 10.3390/cimb46010049

**Published:** 2024-01-17

**Authors:** Maria Consolación Milián-Sorribes, Silvia Martínez-Llorens, David S. Peñaranda, Ignacio Jauralde, Miguel Jover-Cerdá, Ana Tomás-Vidal

**Affiliations:** Aquaculture and Biodiversity Group, Institute of Animal Science and Technology, Universitat Politècnica de València, Camino de Vera s/n, 46022 Valencia, Spain; mamisor@gmail.com (M.C.M.-S.); dasncpea@upvnet.upv.es (D.S.P.); igjaugar@doctor.upv.es (I.J.); mjover@dca.upv.es (M.J.-C.); atomasv@dca.upv.es (A.T.-V.)

**Keywords:** fish oil, fishmeal, *Seriola dumerili*, yellowtail, gut histology, liver histology, microbiota

## Abstract

Fishmeal and fish oil substitution in aquafeeds might have adverse effects on fish growth and health, mainly in carnivorous species, such as Mediterranean yellowtail (*Seriola dumerili*). Mediterranean yellowtail shows great potential as an alternative aquaculture species due to its fast growth and high price on the market, but the need for high-quality protein and fatty acid content in its diets is limiting its production. In order to improve the sustainability of its production, this study was conducted with 360 fish of 35 g to evaluate the effects on fish growth and health. Six diets were used: one control diet without replacement, three with FM replacement (FM66, FM33, and FM0) (33%, 66%, and 100% FM replacement), and two with FO replacement (FO50 and FO0) (50% and 100% FO replacement). The substitution of FM was with vegetable (VM) (corn gluten) and animal (AM) (krill and meat meal) meals. The reductions in FM and FO of up to 33 and 0%, respectively, did not affect the growth and survival of *S. dumerili* at the intestinal morphology level, except for the anterior intestine regarding the lower villi length and width and the posterior intestine regarding the lower width of the lamina propria. On the other hand, the substitution of fish ingredients in the diet affects liver morphology, indicating alterations in the major diameter of hepatocytes or their nuclei. Finally, diet did not affect the gut microbiota with respect to the control, but significant differences were found in alpha and beta diversity when FO and FM microbiota were compared. A 66% FM replacement and total FO replacement would be possible without causing major alterations in the fish.

## 1. Introduction

The cost of feed in aquaculture companies could reach up to 50% of total production costs or even more. Therefore, research and business efforts have aimed to reduce it. The most common solution has been the decrease in fishmeal (FM) and fish oil (FO) content in the diets; nevertheless, this substitution could induce adverse effects on fish growth and health [1,2].

High or complete FM and FO substitutions have been achieved in some trials without a decrement in growth performance, as in juveniles of European sea bass (*Dicentrarchus labrax*) [3], Asian sea bass (*Lates calcarifer*) [4], gilthead seabream (*Sparus aurata*) [5,6], or California yellowtail (*Seriola lalandi*) [7], even in adult yellowtail (*Seriola quinqueradiata*) [8]. Generally, high or complete raw fish ingredients provide negative effects on growth and health status [9]. No formulation has been found that allows 100% substitution of fishmeal in *Seriola* spp., registering lower growth with 100% and 90% of FM substitution in *S. quinquieradiata* [10] and *S. dorsalis* [11], respectively. However, with similar FM substitution (80%) using a mixture of soybean meal, seaweed meal, and squid meal, no effect on growth was reported in *S. rivoliana* [12]. 

When plant-derived meals are used for FM substitution, likely due to the presence of anti-nutritional substances (ANFs), a reduction in nutrient digestibility [13,14,15], alterations at the hepatic/intestinal level, and/or an increase in digestive transit speed [10,16] may occur. Total fishmeal substitutions or up to 75% by a mixture of plant protein in juvenile gilthead seabream reported alterations at the intestinal level, such as a major lamina propria [17] or a smaller absorption surface due to minor villi in alevin barramundi (*Lates calcalifer*) with a 50% fishmeal replacement by soybean [18]. The inclusion of animal meals also provided alterations in intestinal health, such as in adult rainbow trout with a 50% FM replacement by insect meal, which caused a reduction in absorptive epithelial surface, as well as a possible gut inflammation [19]. Negative effects on intestinal histology have been reported with a total FM replacement by animal-derived meals, but other studies with substitution found no effect. In juvenile barramundi, a total substitution of FM by poultry by-product meal resulted in lower digestion and a reduced absorption surface area [20]; however, there was no evidence of histological alterations in the intestine of rainbow trout (*Oncorhynchus mykiss*) with a 60% FM replacement by poultry by-products meal [21] or in Mozambique tilapia (*Oreochromis mossambicus*), with total FM replacement by acid-fermented chicken silage [22]. 

It is anticipated that FM substitution by alternative protein sources (animal and vegetal protein alternatives, hydrolysates, algae, and yeasts) will also affect intestinal microbial composition [9]. In gilthead seabream, total FM substitution by a vegetable diet (soybean, broad bean, wheat, pea, and sunflower) resulted in reported differences at the taxonomic level but not in terms of diversity [23]. Nevertheless, high FM substitution (60%) by vegetal meal (soybean, peas, and canola) decreased the richness and diversity of gut microbiota in rainbow trout [24]. If FM is substituted by animal-derived meals, in juvenile seabream, a minor number of operational taxonomic units (OTUs) and microbial richness [25] was observed when 75% was substituted by meat and bone meal [25]. On the other hand, no differences were found with total substitution by poultry by-product meal [26]. Studies using insect meal for FM replacement have reported an increase in diversity [27,28,29]. Replacing total FM with a mixture of animal and vegetable meals did not alter the gut microbiota in juvenile dusky kob (*Argyrosomus japonicus*) [30].

With FM substitution up to 25% by animal protein blend, at the liver level, negative effects were also observed, registering enlarged hepatocytes in juvenile Japanese sea bass (*Lateolabrax japonicus*) [31] or vacuolization and displacement of nuclei with 60% FM replacement by soybean concentrate in juvenile *Totoaba macdonaldi* [32]. In hybrid grouper (*Epinephelus Fuscoguttatus*♀ *× Epinephelus Lanceolatus*♂), liver alterations were observed after the inclusion of animal protein mixture up to 80%, inducing hepatic steatosis [33]. In alevins seabream, total FM replacement by algae meal and total FO replacement by algae oil showed no differences; all groups looked similar in appearance [34]. Both plant and animal alternative protein sources can have negative effects on the intestinal and liver health of fish [9]. 

On the other hand, if FO is substituted by vegetal oils (VOs), the main problem is the lack of n-3 highly unsaturated fatty acids (HUFAs) in the diets [35]. This is the reason that in marine fish, the use of vegetable oils as a unique lipid source is limited to those species that have a minimum capacity to convert linoleic acid into arachidonic (ARA), and conversion of linolenic acid into EPA and DHA, such as gilthead seabream, cod (*Gadus Morhua*), sea bass, or yellowtail [36]. 

In seawater species, like adult seabream, fish oil substitution of 60% by vegetable oils presented differences in hepatocyte size due to the fat accumulation, displacing the nuclei towards the periphery [37], therefore altering the cellular structure. Conversely, no intestinal and hepatic changes were found in juvenile European sea bass (*Dicentrarchus Labrax*) [38] or juvenile sharp snout seabream (*Diplodus puntazzo*) [39] fed diets with soybean oil up to 60%. 

In studies with fish oil substitution, different effects on fish have been found; in juvenile seabream, a 60% FO substitution by camelina sativa oil did not affect alpha and beta diversity, but a lower abundance of lactic acid bacteria was found, indicating a potentially negative effect on microbiota in gilthead seabream [40]. If the substitution is performed by an oil mixture, the microbiota was split into two groups (Bray–Curtis dendrogram), control and experimental, without affecting richness or diversity [41]. Similar results were found in juvenile European sea bass where very high or complete substitution of FO by a mixture of vegetable oils neither affect the richness and diversity of the intestinal mucosa [3]. The difference in fatty acid composition between vegetable oils and fish oil can influence the function and fluidity of cell membranes, potentially affecting bacterial adhesion and altering the intestinal microbiota profile [41].

In *S. dumerili*, nutritional studies have also been performed to evaluate the effect of FM substitution. Alternative plant protein sources, like soybean, reported lower growth with an FM substitution higher than 50% using soybean [42,43,44]. Nevertheless, with a mixture of vegetal and animal protein sources, it was possible to achieve up to 66% FM replacement [45,46]. Studies in juvenile Mediterranean yellowtail of FO substitution have also been carried out, achieving a total replacement by vegetal oil mixture without effect on growth and survival [47,48]. In other species, such as *S. lalandi*, a total FO replacement by canola oil produced lower growth in juvenile fishes [49], and 90% FM replacement by soy protein concentrate did not have an effect on growth [50]. *S. quinqueradiata* registered similar results since a 25% FM replacement by meal mixture induced lower growth in juvenile fish [51]; meanwhile, a total FO replacement by olive oil did not have an effect [52]. 

At the intestinal level, total or up to 70% FM substitutions reduced the villus height in *S. dorsalis*, and a 20% soybean inclusion revealed a significant reduction in muscular fold number and height [11] or the size of enterocytes and microvilli in *S. dumerili* [44]. The effects of FM replacement on the intestinal microbiome in *S. dumerili* species are scarce. For example, a study of probiotic addition in the diet did not report a relevant effect [53]. In another *Seriola* spp., *S. lalandi*, a comparison among adult wild and aquaculture fish reported differences in bacterial composition and diversity, with major microbiome diversity in wild fishes [54]. When FM was partially replaced, a diet containing 30% soybean meal did not show significant differences [55]; however, in *S. rivoliana*, juvenile fish fed with similar diets exhibited different alpha and beta diversity [56].

Therefore, in this study, in order to achieve the highest level of sustainability, different levels of FM and FO substitution into the diet were assayed in *S. dumerili*, and their possible effects on growth and health status were evaluated.

## 2. Materials and Methods

### 2.1. Fish and Experimental Diets

A total of 360 *S. dumerili* fish from the Futuna Blue S.A. company (Cádiz, Spain) were acclimatized for a month to laboratory conditions before starting the trial. During this time, fish were fed up to apparent satiation twice daily (9:00 a.m.–4:00 p.m.) with a control diet (Table 1), six days a week. Water parameters were constantly monitored: temperature 21.5 ± 2.4 °C, salinity 31 g·L^−1^ (31.5 ± 4.1 g·L^−1^), pH from 7.5 to 8.0, and dissolved oxygen 8 mg·L^−1^. After the acclimatization period, fish were randomly distributed in 18 tanks (20 fish/tank; average weight 38.4 ± 11.6 g). The animals were anesthetized with clove oil at 30 mg·L^−1^, containing 87% eugenol (Guinamas, Valencia, Spain) to limit stress during re-distribution. The trial lasted 154 days. The fish were sampled every 28 days to determine growth and survival data. 

With the aim of evaluating the effect of protein and lipidic substitution in diets, six diets were formulated. Diets were formulated with different levels of fishmeal and fish oil substitution by a mixture of animal and vegetable meal and a mixture of vegetable oils. Diets formulation and composition are shown in Table 1. Diet C had fishmeal as the main protein source and fish oil as the main lipid source. Diets FM66, FM33, and FM0 had FM percentages of 66%, 33%, and 0%, respectively; the rest of the protein percentage was substituted with corn gluten meal, krill meal, and meat and bone meal. Diets FO50 and FO0 had FO percentages of 50% and 0%, respectively; the FO was replaced by a mixture of palm oil and linseed oil (4:1).

### 2.2. Chemical Analysis

Chemical analyses of the dietary ingredients were performed prior to diet formulation and after the fabrication to check the final diet composition. Fish diets ingredients composition were analyzed according to [58] procedures: dry matter, official method 934.01 (105 °C to constant weight); ash, official method 942.05 (incinerated at 550 °C for 5 h); crude protein, official method 990.03 (determined by direct combustion method DUMAS using LECO CN628) and crude lipid, official method 920.39 (extracted with methyl ether using ANKOMXT10 Extractor) The energy was calculated from the C (g) and N (g) balance (GE = 51.8 × C − 19.4 × N) according to [57]. Nitrogen and carbon were analyzed by the Dumas principle (TruSpec CN; Leco Corporation, St. Joseph, MI, USA). 

### 2.3. Histological Analysis

Samples of proximal and distal intestines from three fish per tank were taken at the end of the experiment, fixed in formalin, dehydrated in a different ethanol concentration, and fixed in paraffin. Sections (5 µm) were dyed with PAS-Alcian blue for intestine samples and eosin–hematoxylin for liver samples and observed through light microscopy.

The histological analysis performed was a quantitative analysis. Measurements used a combination of parameters proposed by different authors [17,59,60]. Specifically, the histological analysis focused on the measurement of the length and width of the intestinal villus in the proximal and distal intestine and liver. In gut samples, six villi were measured for each of the three fish collected in each of the three tanks belonging to the same treatment. In addition, the thickness of the lamina propria and the submucosa, muscular, and serous layers were analyzed. In liver samples, six nuclei and six hepatocytes were measured.

All the images of samples were taken with an optical microscope Nikon JAPON 0.90. The images were analyzed using Photoshop CC 2014 software and a conversion into metric units.

### 2.4. 16S rRNA Sequencing and Library Construction

For metagenomic analysis, fish (*n* = 7) were included from those diets with the maximum FO and FM replacement, with the best performance: FM33 and FO0. Genomic DNA (gDNA) extraction was performed from posterior intestinal tract samples using the commercial house DNeasy ^®^ Tissue Kit (QIAGEN, Hilden, Germany). The extracted DNA was evaluated for purity and concentration using a NanoDropTM 2000 spectrophotometer (Thermo Scientific, Milan, Italy). Samples with optical density (OD) readings falling between 1.6 and 1.9 at 260/280 nm were considered acceptable and sent to Macrogen (Seoul, South Korea) for 16S rRNA gene-based metagenomic sequencing. Before sequencing, the quantity of DNA was assessed by processing it through vector 3 fluorometry (Waltham, MA, USA) with the use of DNA-binding dye (Invitrogen, cat. #P7589, Waltman, MA, USA), having a final number of 6 individuals per group. 

The 16S rRNA gene amplicon libraries were generated following the Illumina system protocol “16S Metagenomic Sequencing Library Preparation”. First, following the protocol, PCR—targeting the V3–V4 variable regions of 16S rDNA—was performed using the high-fidelity enzyme Platinum^®^ Taq DNA Polymerase (Thermo Fisher Scientific, Milan, Italy) and the universal primers Pro341F (5′-CCTACGGGNBGCASCAG-3′) and Pro805R (5′-GACTACNVGGGTATCTAATCC-3′) described by [61]. Subsequently, to verify the amplicon size, the samples were run on an Agilent 2100 Bioanalyzer, with the expected size being approximately 550 bp. For the preparation and sequencing of the library, the protocol described by [62] was followed. Briefly, using a kit the PCR products are purified using Agencourt AMPure XP Kit (Beckman Coulter Genomics, Milan Italy) for this purpose. Next, using the Herculase II Fusion DNA polymerase Nextera XT Index Kit V2 (Illumina, San Diego, CA, USA), the Illumina sequencing adapters (P5 and P7) and dual index are ligated to the purified amplicons. 

The libraries were then purified, and the final size of the amplicons was verified using the AMPure XP beads purification system (Beckman Coulter Genomics, Milan, Italy) and Agilent 2200 TapeStation bioanalyzer (Agilent Technologies, Cernuso, Italy). The expected size of the library was approximately 630 bp. Prior to sequencing, libraries were quantified by quantitative PCR (qPCR) using the KAPA Library Quantification Kits for Illumina^®^ platforms (Kapa Biosystems Ltd., London, UK). Finally, libraries pooled in equimolar concentrations are multiplexed and sequenced following the 2 × 300 bp paired-end protocol of the Illumina HiSeq X Ten platform (Illumina, San Diego, CA, USA).

Out of the 15 selected samples (5 from each group), 14 successfully passed the library quality control (QC) assessment. However, one sample from the control group did not meet the QC criteria and was rejected.

### 2.5. Bioinformatics Analysis

The quality of reads was assessed after sequencing of amplicons with FastQC software v.0.11.9 and Qiime2 software v.2021.4 I tools. Trimming and filtering for quality were performed with the q2-demux add-on and subsequent denoising with DADA2 via q2-dada2 [63]. Amplicon sequence variants (ASVs) were aligned with MAFFT via q2-alignment [64] and subsequently used to construct phylogeny with fasttree2 via q2-phylogeny [65]. Taxonomy was assigned to ASVs using the q2 classify-sklearn feature classifier [66] with the naïve Bayes taxonomy classifier itself trained against the Silva 139_99% to 16S V3–V4 region reference sequences [67]. Based on the number of assigned reads, the relative abundances (%) of taxa in each gut microbiota sample were calculated. 

In addition, the diversity between serials fed the same experimental diets was evaluated, as well as the variability existing between diets C, FM33, and FO0. For this purpose, the following alpha diversity indices, which evaluate taxonomic richness and abundance, were determined: Shannon index, Chao1 index, Evenness index, Simpson index, and Berger–Parker index. The alpha rarefaction curve, observed features (number of observed taxa), and Faith’s phylogenetic diversity index have also been determined. On the other hand, the differences between samples of both groups have been analyzed by beta diversity. For this purpose, UniFrac, Unweighted UniFrac (presence/absence matrix), and Weighted UniFrac (presence/absence/abundance matrix) analyses were performed, from which principal coordinate analysis (PCoA) plots of three dimensions were constructed. All diversity indices were obtained using Qiime2 software v.2021.4 [66].

### 2.6. Statistical Analysis

Growth data and histological analysis were treated using one-way analysis of variance (ANOVA). The Newman–Keuls test was used to assess specific differences among diets at 0.05 significant levels (Statgraphics, Statistical Graphics System, Version Centurion XVI, Warrenton, VA, USA).

## 3. Results

### 3.1. Growth Data

**Figure 1:**
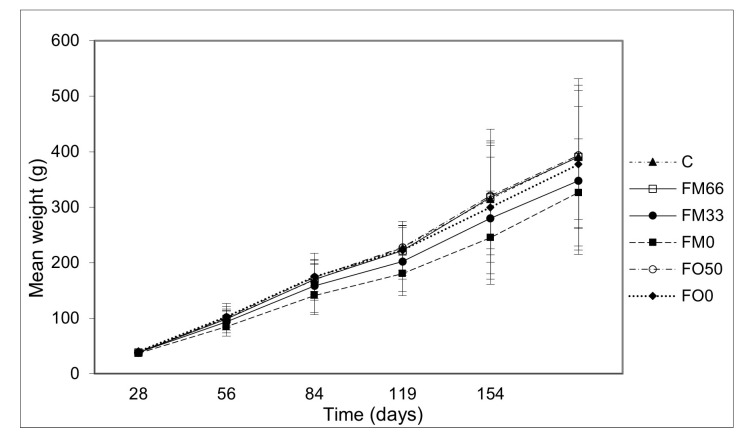
Mean weight evolution of fish per treatment along the growth trial.

For 154 days of the trial, no negative effects were found, neither in partial nor complete fish oil substitution (Figure 1); lower values were observed in fish fed with FM0 diet but without statistical differences.

**Figure 2:**
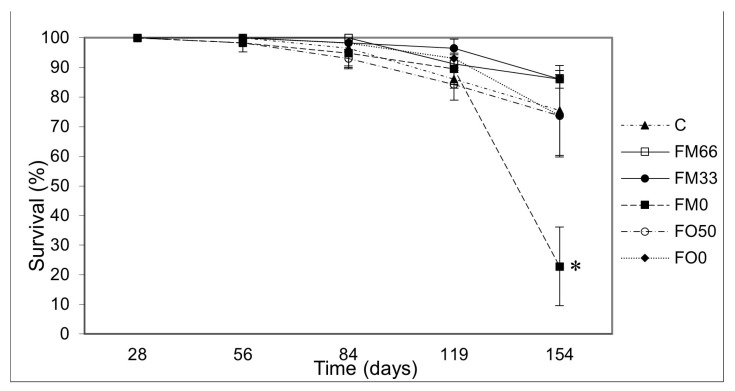
Survival per treatment during the growth trial. The asterisk indicates significant differences between treatments (*p* < 0.05).

A complete fishmeal substitution also affected the survival at the end of the trial, with a lower percentage (22.8 ± 13%) with respect to control and other experimental diets (Figure 2).

### 3.2. Histological Analysis

At the anterior intestine level, fishmeal replacement reduced the length and width of the villi and the thickness of the muscular layer (Table 2). At the posterior level, wider and narrower lamina propria and muscle layer, respectively, were registered in the experimental groups with higher fishmeal substitution (Table 2 and Figure S1).

Regarding liver histology, FM replacement decreased the diameter of the hepatocyte (Table 3 and Figure S2). 

On the other hand, at the anterior level, FO replacement affected the width of lamina propria but without a clear effect, with lower values in FO50 and higher in the FO0 group (Table 4). Nevertheless, at the posterior level, the substitution induced a clear reduction in the width of lamina propria (Table 4 and Figure S3). At the posterior level, differences were also found in the width villi, but again without a clear effect in relation to FO substitution. 

At the liver level, FO substitution did not affect the diameter of hepatocytes; instead, a reduction in nuclei was observed with respect to control diets (Table 5 and Figure S4). 

### 3.3. Gut Microbiota

After quality filtering, a total of 1,028,047 reads were obtained, with reads per sample ranging from 41,952 to 63,319. The mean of the percentage of non-chimeric merged sequences was 82.37 ± 3.05%. A total of 17 phyla and 34 genera were identified. From the 17 phyla identified in *Seriola dumerili* intestinal gut, more than 94% of operational taxonomic units belonged to six of them. The most abundant phyla (≥5%) common to all fish analyzed were *Spirochaetes, Proteobacteria*, and *Actinobacteria*. 

Based on the diet, we registered slight differences (Figure S5). Control diet fish were dominated by the phylum *Proteobacteria* (57 ± 13.3%), followed by *Spirochaetes* (16 ± 12.1%), and *Bacterioidetes* (10 ± 4.5%). Within the phylum *Proteobacteria,* the most numerous genera were *Legionella* (7.1%), *Rhodobacterium* (4.9%), and *Sphingomona* (3%). The *Brevinema* spp. (15.6%) of the phylum *Spirochaetota* also stands out. Other genera with less representation are *Corynebacterium* (1.8%), *Staphylococcus* (1.75%), and *Mycobacterium* (1.4%). 

The FM33 diet showed a similar dominance: *Proteobacteria* (61 ± 12%) followed by *Spirochaetes* (22 ± 12%), but the third most abundant was *Actinobacteria* (10 ± 5.8%). At the genus level, the highest representation corresponds to *Brevinema* (21.9%), followed by *Legionella* (6.75%), *Vibrio* (6%), *Rhodobacterium* (4.3%), *Marivita* (3.9%), and *Sphingomona* (2.4%). The *Mycobacterium* spp. from the *Actinobacteriota* phylum also has an important presence (9.4%).

If FO was totally replaced (FO0), the intestinal microbiota was also dominated by *Proteobacteria* (62 ± 13.3%), with the following genera standing out: *Rhodobacterium* (7.3%), *Sphingomona* (5.2%), *Legionella* (2.5%), and *Photobacterium* (3.4%). *Firmicutes* were represented by 13 ± 1.5% and *Bacterioidetes* by 9 ± 5.1%. In the phylum *Firmicutes*, the genera *Staphylococcus* (3.6%) and *Thermicanus* (2%) were also abundant. Other genera with lower representation are *Brevinema* (2.6%), *Mycobacterium* (1.4%), *Atopobium* (1.6%), and *Lactobacillus* (2.6%).

Significant differences were found for *Cyanobacteria*, with lower values in the fish fed FM33. Although no significant differences were found in the rest of the phyla, perhaps due to the high intra-group variability, with FM replacement, the phyla *Bacteroidetes* and Firmicutes show a downward tendency, while the phyla *Actinobacteria, Proteobacteria, Spirochaetes*, and *Dependentiae* have a tendency to increase. When FO is substituted, the relative abundance of *Spirochaetes* and *Actinobacteria* tends to decrease and *Firmicutes* and *Cyanobacteria* to increase. In the case of the analysis of bacteria at the genus level, no differences were found, quite possibly the same as at the phylum level due to the variability in the groups.

**Figure 3:**
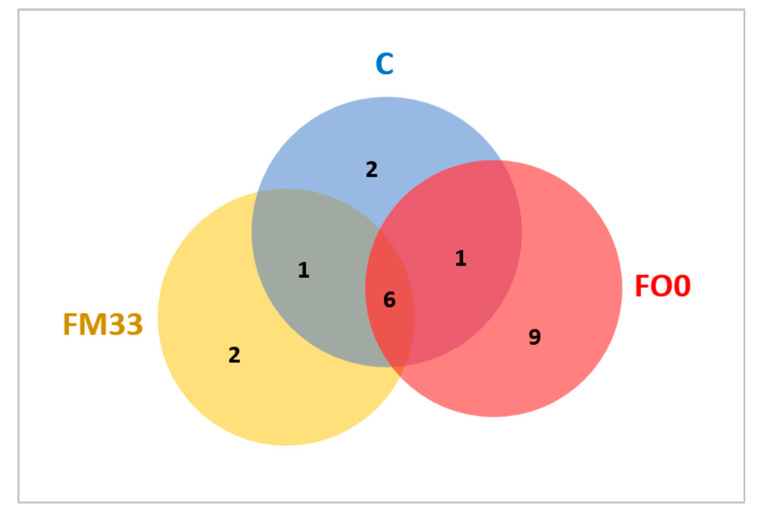
Venn diagrams at the genus taxonomic level. OTUs included were present in percentages above 1%. Common bacterial genera are displayed in the middle regions, and specific bacterial families of fish fed the C, FM33, and FO0 diets are displayed in blue, orange, and red colors.

**Figure 4:**
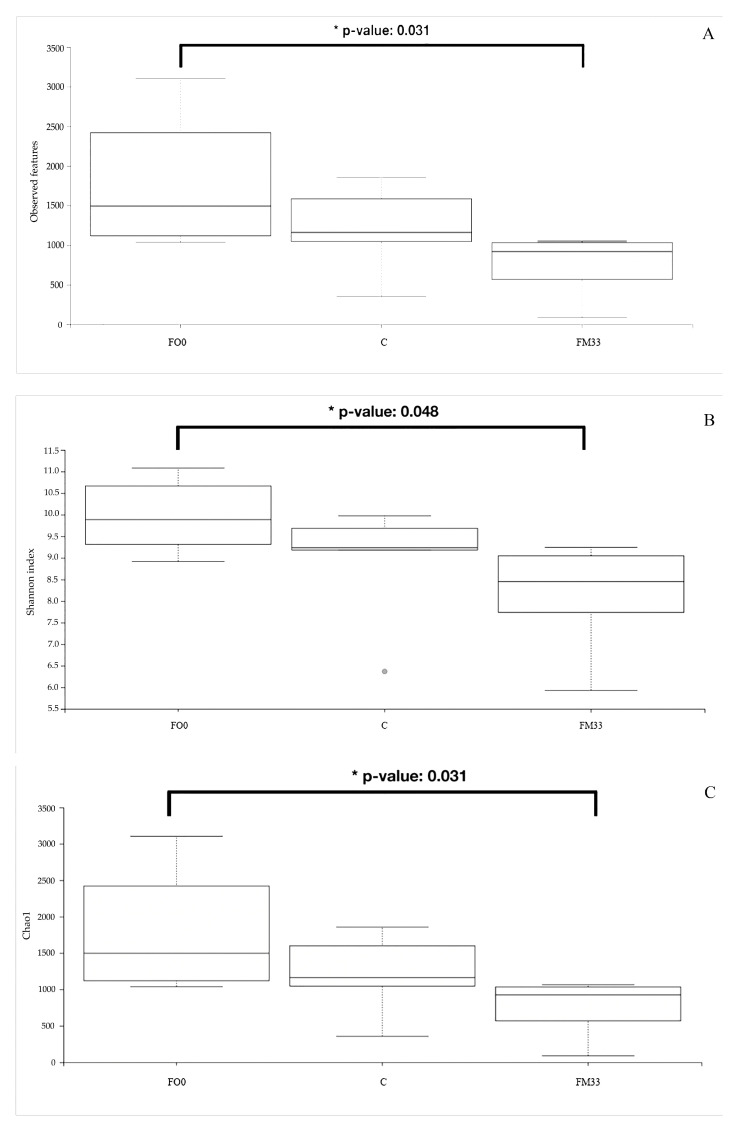
α-diversity measures: (**A**) observed features, (**B**) Shannon index, and (**C**) Chao1 index of 16S microbiota gut community compositions of experimental groups control diet (**C**), the FM substitute diet (FM33), and FO replacement (FO0). The asterisk indicates significant differences between treatments (*p* < 0.05).

**Figure 5:**
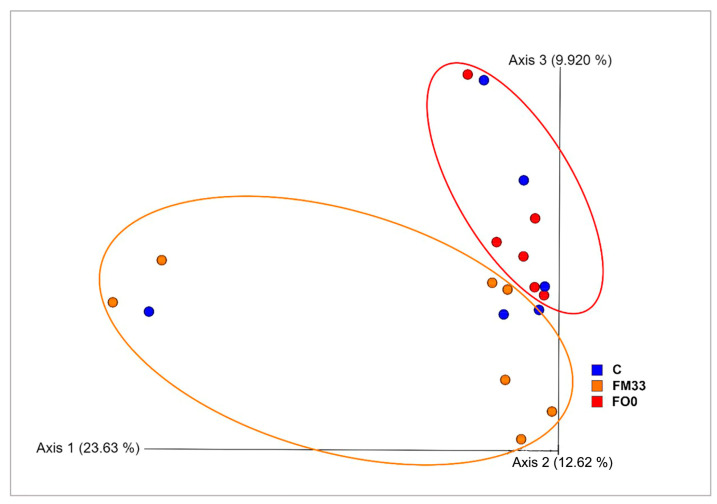
PCoA Unweighted Unifrac distance of the microbiota of experimental groups: control diet (C), FM substitute diet (FM33), and FO replacement (FO0). The first, second, and third principal components explained 46% of the sample variations.

Thanks to the Venn diagram, a broader perspective is shown (Figure 3). A core of six bacterial genera was shared by three experimental groups. Only one genus was common between FM33 and the control diet (*Babeliaceae*), and the other between the control and FO0 diets (*Staphylococcus*). A greater number of genera specifically associated with the diet FO0 were found. *Photobacterium, Enhydrobacter, Thermicanus Atopobium*, *Lactobacillus, Prevoleta, Neisseria*, and *Muribaculaceae* genera were exclusive to FO0, while *Cloacibacterium* and *Corynebacterium* were only observed in fish fed C and *Marivita* and *Babeliae* in FM33. Finally, significant differences in α-diversity were found between FM33 and FO0 but not with the control group (C). Fish fed the FO0 diet not only had a higher number of taxa observed than FM33 (Figure 4A) but also major species richness using Chao1 (Figure 4B) and Shannon (Figure 4C) indices. On the other hand, surprisingly, the control diet presented middle values without differences in either FO0 or FM33. β-diversity was visualized using a PCoA Unweighted Unifrac distance plot, and significant separation of the gut microbial compositions by treatment was found (Figure 5). The samples from the FO0 diet were grouped and separated from the FM33 samples.

## 4. Discussion

Attending to the results, it could be confirmed that high fishmeal and total fish oil substitution by a mix of alternative vegetal and animal sources is possible without affecting the growth and health of *S. dumerili*. Nevertheless, a total fishmeal substitution reported higher mortality. Previous work with total FM substitution registered similar results, likely as a consequence of the presence of antinutrients in plant protein sources. It has been reported that antinutrients may cause immune depression events [13] and nutrient deficiencies [68], leading to a deterioration of intestinal health, increasing the susceptibility to pathogens, and finally to death [2,69]. These results are in discordance with previous studies in juvenile *Seriola dumerili*, when soybean was used to replace FM by up to 50%, and lower growth was obtained, but without affecting survival [42,43]. In this trial, the FM replacement affected survival but did not affect growth.

As has been described, the poor results obtained with a total FM substitution may be due to the fact that plant protein sources induce intestinal inflammation [9,70]. This inflammatory response is characterized in salmonids by a shortening of mucosal folds and a widening of lamina propria [60] due to the infiltration of inflammatory cells identified as lymphocytes, macrophages, eosinophils and cells, granular neutrophils, and diffuse immunoglobulin M (IgM) [71]. However, the inclusion of plant protein sources does not produce such striking alterations in other species as seabream [72], and there is no available information about the effect on Mediterranean yellowtail, but it has been shown that high FM replacement (75%) can cause a decrease in enterocytes and villi size at intestine level [44]. In this work, the results showed significant differences in the length of the villi between the different diets, mainly in the anterior intestine, but it cannot be stated that the surface has been reduced with the substitution since it also depends on the number of villi. This is contrary to what was observed in perch [73] and turbot [74], in which differences were found in the posterior intestine with a shortening of villi when FM was substituted by soybean and poultry by-product meal. In the posterior intestine, a thickness increase in lamina propria (LP) was observed with FM substitution; in fact, the FM0 group registered the thickest LP, close to enteritis symptom. Similar results were reported in turbot, where the replacement of fishmeal caused enteritis in the posterior intestine [74]. FO substitution also induced LP differences in both the anterior and posterior intestines, registering major values. In previous studies, similar data were observed in this species [47], likely caused by an inflammatory process since the LP width is usually related to the cell number that forms it and the level of eosinophilic cell infiltration.

An inadequate diet can cause hepatocyte vacuolization, fat degeneration, metabolic changes, or necrosis [75,76]. The alterations found can be different depending on whether the replacement is proteinic or lipidic. Smaller hepatocyte diameter was observed with FM substitution in this work, which is a hallmark of hepatic apoptosis, usually considered an indicator of unbalanced nutrition [9]. Similar results were obtained in *Ameiurus nebulosus* [77] and *Sparus aurata* [78], where a complete FM replacement led to the appearance of inflammatory signs and irregular hepatocytes with smaller nuclei.

In this work, FO substitution caused a decrease in the hepatocyte size and nuclei, which is also considered an indicator of fish malnutrition. In fish fed with vegetable oils, similar results were found in gilthead seabream [79] or Japanese sea bass [80] when a 60% FO replacement resulted in steatosis and the appearance of vacuoles in the hepatocyte. A higher hepatocyte size by leukocyte infiltration and a reduction in its nuclei have also been reported as indicators of liver damage, possibly due to the presence of anti-nutritive substances in the diet [79]. If we attend to the visual aspect of the liver after FM substitution (Figure S3), a significant presence of fat can be observed, especially in the fish fed with the control feed with respect to experimental diets. The low number of vacuoles may be related to lower enzyme activity [79].

Microbial communities are also a tool to evaluate intestinal health status [81]. In fact, an association has been established between a high richness and relative abundance in the gastrointestinal tract with good metabolic capacity and animal welfare [82]. A substitution of traditional protein sources for alternative plant or animal sources ought not to cause a negative effect on gut microbial communities [24]. In fact, a moderate fishmeal substitution did not report a significant effect on microbiota diversity and composition in seabream [83]; however, higher or complete substitutions of FM can affect the immune system [69], causing inflammatory secretions [84] or enteropathy [85]. As far as is known, no studies have been conducted to date on the influence of fish oil and fishmeal substitution on the gut microbiota of *Seriola dumerili*.

In this work, the experimental groups with the highest FO and FM substitution with no significant differences in terms of growth and survival with respect to the control group were chosen to evaluate their microbiota, with the aim of confirming that no relevant alterations have been induced by the fish source replacement.

Regardless of diet, in this study, the dominant phylum was *Proteobacteria*, as in other species: salmon [86], seabream [2,87], carp (*Cyprinus carpio*) [88], or trout [24]. In previous studies in *Seriola lalandi*, farmed fish registered *Firmicutes* as the dominant phylum; meanwhile, in wild fish, it was *Proteobacteria* [54,89]. In fact, *Proteobacteria* is usually the dominant phylum in animals of non-herbivorous trophic levels [90]. In the control diet, the most relevant classes were *Gammaproteobacteria* and *Alphaproteobacteria*, which are relevant for adaptation to environmental changes [56]. In particular, *Gammaproteobacteria* are key in animal nutrition and maintenance of other beneficial bacteria [91]. On the other hand, the *Photobacterium* spp. of the *Vibrionaceae* family was over-represented in Mediterranean yellowtail after fish oil replacement. In this work, *Photobacterium* was only found in representative amounts in FO0. These genera have also been associated with fish welfare as a result of a mutualistic relationship with their host for chitin digestion, but also, in some cases, with pathogens and producers of enzymes such as neuraminidases that can cause tissue damage [92,93]. Other abundant phyla identified in the control diet were *Bacteroidota*, *Actinobacteriota*, and *Firmicutes*, coinciding with those reported in *S. rivoliana* [56] and *S. lalandi* [54,94]. The latter two are phyla related to carbohydrate and polysaccharide metabolism [95,96]. However, when dietary fishmeal is substituted, the abundance of *Actinobacteria* increases, especially the *Mycobacterium* spp., which could be due to the fact that dietary needs have not been adequately met, which is known for its role as a producer of secondary metabolites in the gut [91]. Nevertheless, the opposite case occurs in FO substitution in which the presence of *Actinobacteriota* decreases; although the genus *Atopobium* is only found in fish fed without FO, *Atopobium* spp. has the ability to cause periodontitis, vaginitis, and urethritis, as well as bacteremia and sepsis [97]. Furthermore, the presence of the class *Ruminococcaceae* in fishes fed FM33 and FO0 may be related to the production of butyrate from non-digestible complex polysaccharides, which is known to benefit host physiology, improving the ability to obtain energy from the diet, reinforcing the intestinal epithelial barrier and modulating immune function [98]. When fishmeal was replaced, a relatively high abundance of *Brevinema* spp. was observed, decreasing considerably in fish fed without FO. Although some species are known to cause disease in vertebrates, they are also known as endosymbionts, participating in lignocellulose digestion and nitrogen fixation in termites [99]. In addition, the enrichment of *Brevinemia* and other species of the family *Spirochaetaceae* in salmon has recently been linked to the expression of genes involved in immune response and distal gut barrier function [27]. Similarly, bacteria of the phylum *Spirochaetes* are involved in the fermentation of carbohydrates, transporting non-digestible sugars across their cell membranes [100]. In fish fed with high levels of dietary fish oil, the abundance of the *Bacilli* class, which dominates the phylum *Firmicutes*, is notable. Although there are species that might cause disease, *Bacillus* spp. is being used to improve water quality [101] or as probiotics to improve growth and immune status [102,103,104,105]. On the other hand, the increase in cyanobacteria in fish fed FO0, significantly higher than FM33, may be relevant since it has been demonstrated to produce metabolites that alter nutrient uptake [106].

According to α- and β-diversity analysis, a clear separation was observed among the FO0 and FM33 groups; meanwhile, the control diet registered a middle value without significant differences with FM33 and FO0. Like in this work, in *Seriola lalandi* or gilthead seabream, a minor diversity was reported after FM substitution by plant protein as an alternative to animal protein sources [25,94]. Similar results can also be found in largemouth bass (*Micropterus salmoides*) using algae meal [107] or Nile tilapia (*Oreochromis niloticus)* using rice protein concentrate [108]. However, in salmon, a complete FO substitution did not alter the intestinal bacterial population using a mix of rapeseed, linseed, and palm oils [109]. In Nile tilapia, algae oil inclusion does not affect alpha microbiome diversity either [110]. In this study, no differences were found with respect to the control diet, but there were differences in those fish fed with high fishmeal substitution, which is in concordance with previous studies detailed above.

In summary, it can be concluded that a total FO and high FM substitution is possible without affecting the growth performance and has no relevant effect on intestinal histology and microbiota. These results suggest a robust tolerance of the Mediterranean yellowtail to the tested levels of fishmeal and fish oil substitution, highlighting the potential for sustainable aquaculture practices without compromising intestinal health and microbial balance. Nevertheless, the studies should continue in order to find more sustainable raw materials to include in the diets without affecting growth performance and intestinal health.

## Figures and Tables

**Table 1:** Formulation and proximate composition of the experimental diets.

	C	FM66	FM33	FM0	FO50	FO0
Ingredients (g/Kg)						
Fishmeal ^1^	525	350	175		525	525
Wheat meal ^2^	235	108	43		235	235
Wheat gluten ^3^	130	130	140	180	130	130
Corn gluten ^4^		100	100	100		
Defatted Krill ^5^		120	230	345		
Meat Meal ^6^		80	198	250		
Linseed oil					9	18
Palm oil					36	72
Fish oil	90	92	88	95	45	0
L-Methionine			3	5		
L-Lysine Clh			3	5		
^v^ Multivitamin and minerals mix	20	20	20	20	20	20
Analyzed composition (g/kg dry matter)						
Dry matter (DM)	888	888	895	902	894	899
Crude protein (CP)	530	580	604	633	534	540
Crude lipid (CL)	139	142	138	137	133	135
Ash	103	106	121	115	87	87
Mainly Amino acids ^a^ (g/kg dry matter)						
Arginine	35.1	31.9	37	34.5	34.8	34.6
Leucine	25.8	27.4	27.1	26.7	26	25.9
Lysine	33.3	29.2	32.1	28	32.9	33
Methionine	11	9.9	11.6	12.5	11.1	11.4
Mainly Fatty Acids ^b^ (g/kg dry matter)						
EPA	19.65	17.74	15.07	13.23	13.97	7.63
DHA	15.33	12.85	10.52	8.97	10.91	7.08
ARA	1.01	0.87	0.74	0.62	0.73	0.39
LC-PUFA ^c^	48.31	44.97	41.08	38.27	34.44	20.31
Calculated values						
^µ^ Energy (kJ/g)	23.8	24.1	23.7	23.8	24.5	24.0

Note: Diet formulations presented in this table have been previously published in [45,48]. ^1^ Fishmeal (93.2% DM, 70.7% CP, 8.9% CL, 15.1% ash). ^2^ Wheat meal (92.4% DM, 17.1% CP, 2.4% CL, 78.3% CHO, 2.4% ash). ^3^ Wheat gluten (93.3% DM, 8.1% CP, 9% CL, 73.9% CHO, 9% ash). ^4^ Corn gluten (93.3% DM, 72.9 CP, 0.9% CL, 25.3% CHO, 0.9% ash). ^5^ Extracted krill meal: product obtained by removing the fat with ethanol (87.8% DM, 69.7% CP, 2.9% CL, 8.17% CHO, 11.6% ash); VALGRA S.A. Beniparrell. Valencia. Spain. ^6^ Meat and bone meal (97.0% DM, 53.1% CP, 15.3% CL, 4.7% CHO, 26.9% ash, 17.69 kJ−1 energy); VALGRA S.A. Beniparrell. Valencia. Spain. ^v^ Multivitamin and minerals mix (values are g/kg except those in parentheses): premix: 25; Choline, 10; DL-a-tocopherol, 5; ascorbic acid, 5; (PO_4_)_2_Ca_3_, 5. Premix composition: retinol acetate, 1,000,000 IU/kg; calciferol, 500 IU/kg; DL-a-tocopherol, 10; menadione sodium bisulfite, 0.8; thiamine hydrochloride, 2.3; riboflavin, 2.3; pyridoxine hydrochloride, 15; cyanocobalamin, 25; nicotinamide, 15; pantothenic acid, 6; folic acid, 0.65; biotin, 0.07; ascorbic acid, 75; inositol, 15; betaine, 100; polypeptides, 12. ^a^ Total amino acids values in [47]. ^b^ Total fatty acids values in [46,48]. ^c^ LC-PUFA: long-chain polyunsaturated fatty acids. ^µ^ Energy (%) = (51.8 × (%C/100)) − (19.4 × (%N/100)). Calculated according to [57].

**Table 2:** Histological measurements of the anterior and posterior intestines in fish fed the experimental diets (FM replacement) at the end of the growth trial.

	C	FM66	FM33	FM0	*p*-Value
*Anterior intestine*					
SL (μm)	48 ± 4	43 ± 4	52 ± 4	58 ± 4	0.083
ML (μm)	147 ^b^ ± 10	109 ^a^ ± 9	140 ^b^ ± 10	165 ^b^ ± 10	0.001
SML (μm)	83 ± 5 ^b^	63 ± 4 ^a^	73 ± 5 ^ab^	69 ± 5 ᵃᵇ	0.015
VL (μm)	661 ± 46 ^b^	467 ± 46 ^a^	507 ± 47 ^a^	430 ± 44 ^a^	0.003
WVL (μm)	64 ± 4 ^b^	61 ± 4 ^ab^	57 ± 4 ^ab^	48± 4 ^a^	0.035
WLP (μm)	15.6 ± 1.3	16.3 ± 1.3	15.9 ± 1.3	15.3 ± 1.2	0.946
GC	60 ± 5	47 ± 5	53 ± 5	47 ± 5	0.221
*Posterior intestine*					
SL (μm)	48 ± 4	48 ± 4	58 ± 5	56 ± 4	0.263
ML (μm)	117 ^ab^ ± 8	113 ^a^ ± 8	147 ^b^ ± 10	148 ^b^ ± 9	0.007
SML (μm)	67 ± 4	64 ± 4	77 ± 5	60 ± 5	0.140
VL (μm)	510 ± 37	594 ± 29	525 ± 54	494 ± 39	0.169
WVL (μm)	60 ± 3	63 ± 2	55 ± 4	62 ± 3	0.444
WLP (μm)	11.1 ^a^ ± 1.2	11.1 ^a^ ± 1.7	15.5 ^a^ ± 1.1	19.8 ^b^ ± 1.3	0.001
GC	43 ± 4	49 ± 3	42 ± 6	51 ± 4	0.379

The values represent the mean ± standard error (n = 18). Different superscript letters indicate significant differences between treatments (*p* < 0.05). SL: thickness of serosa layer; ML: thickness of muscular layer; SML: thickness of submucosa layer; VL: villus length; WVL: width of villi; WLP: width of *lamina propria*; GC: goblet cell.

**Table 3:** Histological measurements of liver in fish fed the experimental diets (FM replacement) at the end of the growth trial.

	C	FM66	FM33	FM0	*p*-Value
Nuclei diameter (μm)	59 ± 1	61 ± 1	57 ± 1	62 ± 2	0.432
Hepatocyte diameter (μm)	152 ^b^ ± 3	148 ^b^ ± 3	141 ^ab^ ± 4	130 ^a^ ± 6	0.007

The values represent the mean ± standard error (n = 54). Different superscript letters indicate significant differences between treatments (*p* < 0.05).

**Table 4:** Histological measurements of the anterior and posterior intestines in fish fed the experimental diets (FO replacement) at the end of the growth trial.

	C	FO50	FO0	*p*-Value
*Anterior intestine*				
SL (μm)	48 ± 4	55 ± 4	55 ± 4	0.239
ML (μm)	147 ± 10	151 ± 9	174 ± 10	0.076
SML (μm)	83 ± 5	84 ± 4	88 ± 5	0.722
VL (μm)	661 ± 45	541 ± 51	542 ± 61	0.148
WVL (μm)	64 ± 4	50 ± 5	65 ± 6	0.057
WLP (μm)	15.6 ^ab^ ± 1.4	12.3 ^a^ ± 1.6	18.3 ^b^ ± 1.9	0.049
GC	60 ± 5	48 ± 5	48 ± 7	0.166
*Posterior intestine*				
SL (μm)	48 ± 4	45 ± 4	47 ± 4	0.864
ML (μm)	117 ± 8	128 ± 8	134 ± 8	0.385
SML (μm)	67 ± 4	65 ± 4	69 ± 4	0.811
VL (μm)	510 ± 37	433 ± 35	498 ± 49	0.201
WVL (μm)	60 ^ab^ ± 3	51 ^a^ ± 3	64 ^b^ ± 4	0.030
WLP (μm)	11.1 ^a^ ± 1.2	10.4 ^a^ ± 1.2	15.3 ^b^ ± 1.7	0.005
GC	43 ± 4	33 ± 4	42 ± 5	0.108

The values represent the mean ± standard error (n = 18). Different superscript letters indicate significant differences between treatments (*p* < 0.05); Newman–Keuls test. SL: thickness of serosa layer; ML: thickness of muscular layer; SML: thickness of submucosa layer; VL: villus length; WVL: width of villi; WLP: width of lamina propria; GC: goblet cell.

**Table 5:** Histological measurements of the liver in fish fed the experimental diets (FO replacement) at the end of the growth trial.

	C	FO50	FO0	*p*-Value
Nuclei diameter (μm)	59 ^a^ ± 1	54 ^b^ ± 1	56 ^ab^ ± 2	0.011
Hepatocyte diameter (μm)	152 ^a^ ± 3	133 ^b^ ± 3	142 ^b^ ± 4	0.001

The values represent the mean ± standard error (n = 54). Different superscript letters indicate significant differences between treatments (*p* < 0.05); Newman–Keuls test.

## Data Availability

Data is contained within the article and Supplementary Material.

## Supplementary Materials

The following supporting information can be downloaded at: https://www.mdpi.com/article/10.3390/cimb46010049/s1. Figure S1: microphotographs of posterior intestine section of yellowtail: (a) C (20×), (b) FM66 (20×), (c) FM33 (20×), and (d) FM0 (20×); hematoxylin and eosin staining. Figure S2: microphotographs of posterior intestine section of yellowtail: (a) C (20×), (b) FO50 (20×), and (c) FO0 (20×); hematoxylin and eosin staining. Figure S3: microphotographs of liver section of yellowtail: (a) C (20×), (b) FM66 (20×), (c) FM33 (20×), and (d) FM0 (20×); hematoxylin and eosin staining. Figure S4: microphotographs of liver section of yellowtail: (a) C (20×), (b) FO50 (20×), and (c) FO0 (20×); hematoxylin and eosin staining. Figure S5: 16S OUT relative abundance (%) of the main taxa at the phylum level present in the control diet (C), the FM substitute diet (FM33), and FO replacement (FO0).
