# Supplementary material for: Growth, Survival, and Intestinal Health Alterations in Mediterranean Yellowtail (Seriola dumerili) Due to Alternatives to Fishmeal and Fish Oil
Source: Curr Issues Mol Biol. 2024 Jan 17;46(1):753–72. doi: 10.3390/cimb46010049 (PMC10814527; doi:10.3390/cimb46010049)
Supplement: Supplementary file 1 [file cimb-46-00049-s001.zip › cimb-2778204-supplementary.pdf]

Supplementary materials

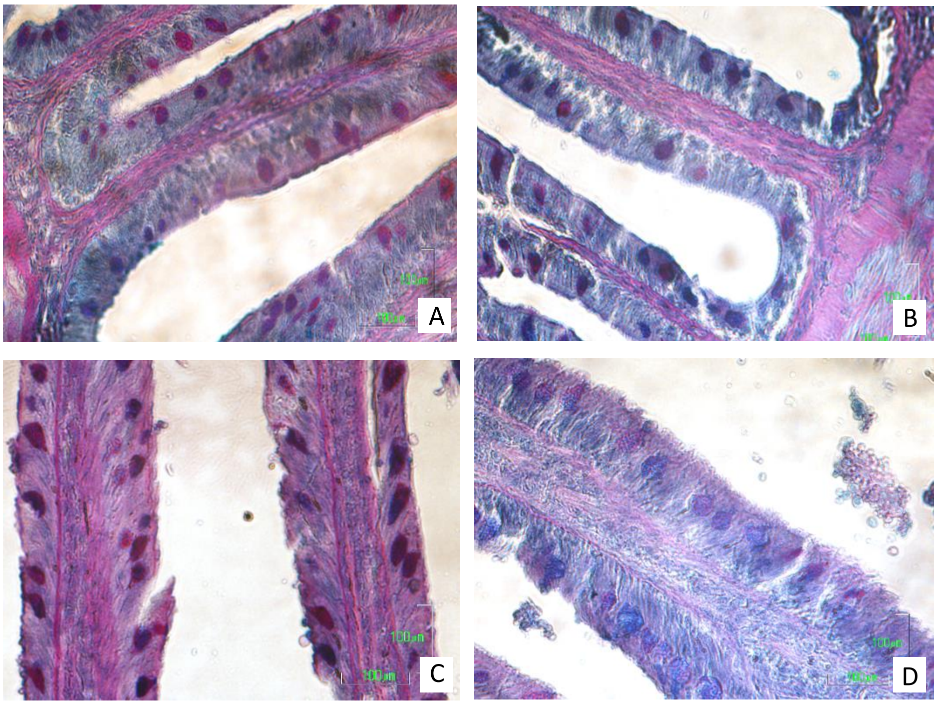

Figure S1: Microphotographs of posterior intestine section (a) C (20×). (b) FM66 (20×). (c) FM33 (20×). (d) FM0 (20×) of yellowtail. Haematoxylin and eosine staining.

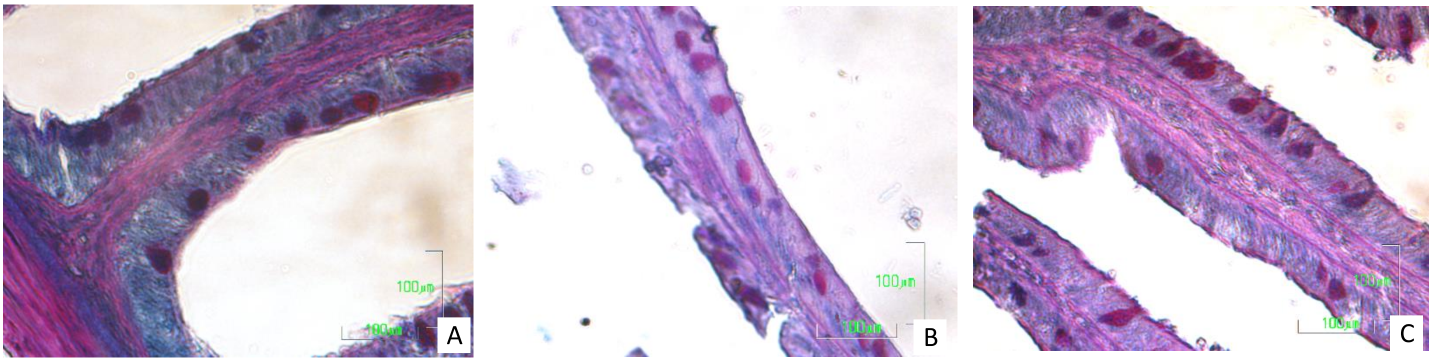

Figure S2: Microphotographs of posterior intestine section (a) C (20×) (b) FO50 (20×) (c) FO0 (20×) of yellowtail. Haematoxylin and eosine staining.

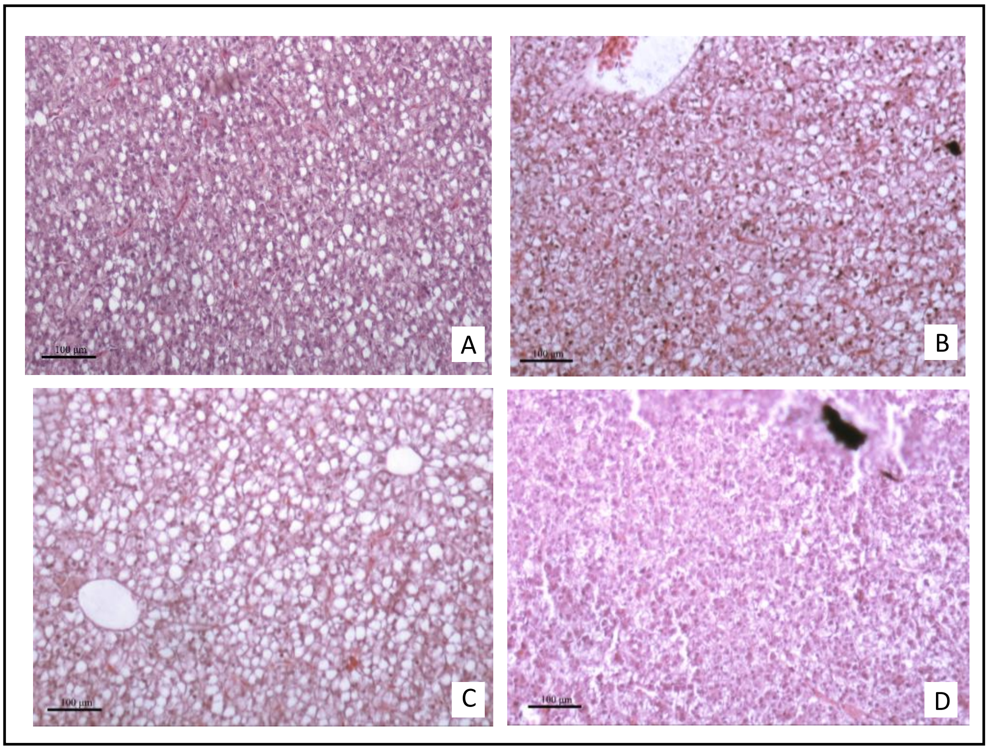

Figure S3: Microphotographs of liver section (a) C (20×). (b) FM66 (20×). (c) FM33 (20×). (d) FM0 (20×) of yellowtail. Haematoxylin and eosine staining.

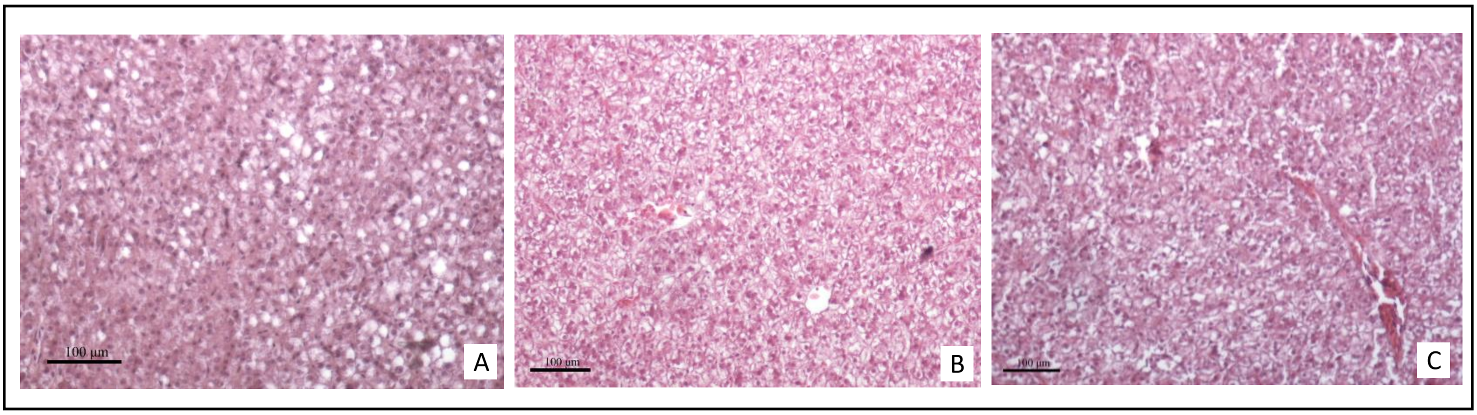

Figure S4: Microphotographs of liver section (a) C (20×) (b) FO50 (20×) (c) FO0 (20×) of yellowtail. Haematoxylin and eosine staining.

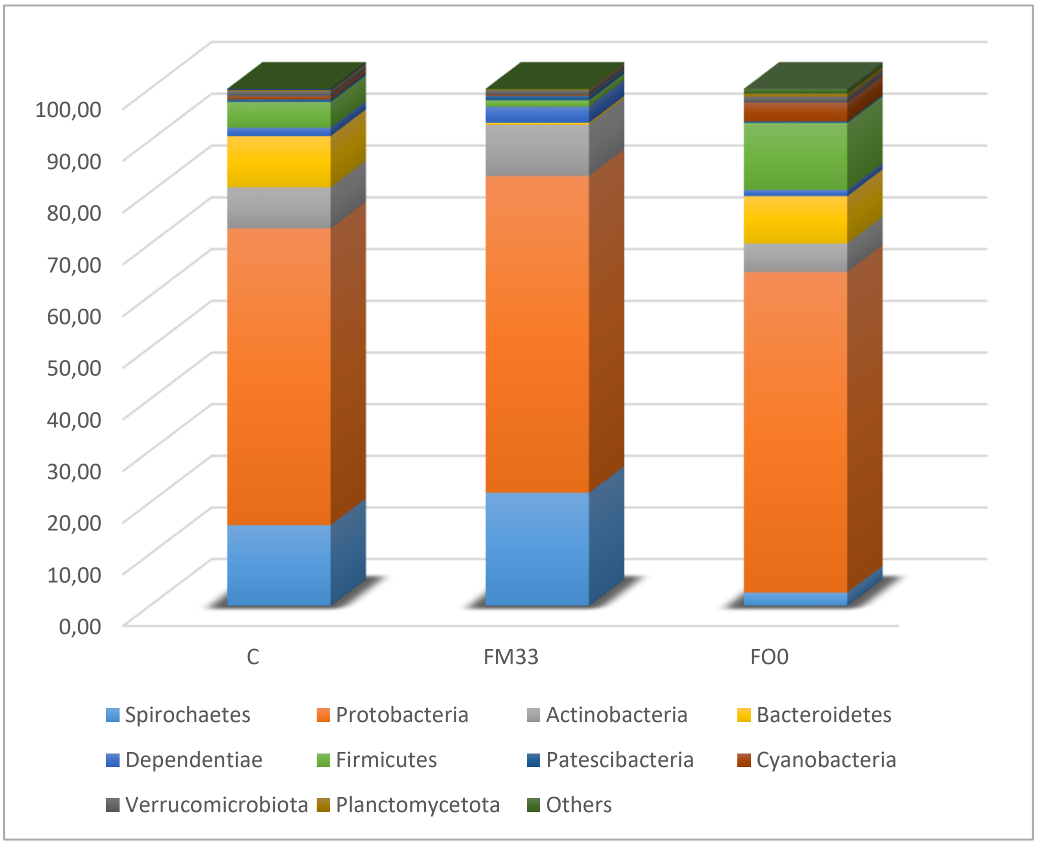

Figure S5. 16S OUT Relative abundance (%) of the main taxa at the phylum level present in control diet (C), the FM substitute diet (FM33) and FO replacement (FO0).
